# Avian influenza in Latin America: A systematic review of serological and molecular studies from 2000-2015

**DOI:** 10.1371/journal.pone.0179573

**Published:** 2017-06-20

**Authors:** Alejandra Afanador-Villamizar, Carlos Gomez-Romero, Andres Diaz, Julian Ruiz-Saenz

**Affiliations:** 1 Semillero de Investigación en enfermedades Infecciosas - InfeKto, Universidad Cooperativa de Colombia, Bucaramanga, Colombia; 2 PIC - Pig Improvement Company LATAM, Querétaro, Mexico; 3 Grupo de Investigación en Ciencias Animales GRICA, Universidad Cooperativa de Colombia, Bucaramanga, Colombia; Icahn School of Medicine at Mount Sinai, UNITED STATES

## Abstract

Avian influenza or bird flu is a highly contagious acute viral disease that can occur in epidemics and cross-border forms in poultry and wild birds. The characteristics of avian influenza viruses (AIVs) allow the emergence of new viral variants, some with zoonotic and pandemic potential. AIVs have been identified in Latin America; however, there is a lack of understanding of these viruses at the regional level. We performed a systematic literature review on serological or molecular evidence of AIVs circulation in Latin America. Methods were designed based on the PRISMA and STROME guidelines. Only peer-reviewed studies published between 2000 to 2015 and data was analysed based on country, viral subtype, avian species, and phylogenetic origins. From 271 studies initially found only twenty-six met our inclusion criteria. Evidence of AIVs infection was found in most Latin American countries, with Mexico as the country with the largest number of conducted studies and reported cases during the period analysed, followed by Chile and Argentina. Most of the AIVs were early reported through surveillance systems and at least 14 different subtypes of influenza viruses were reported in birds, and the presence of both low (92.9%) and high (7.1%) pathogenic AIVs was shown in Latin America. Of the reported AIVs in Latin America, 43.7% belong to migratory birds, 28.1% to local wild birds, and 28.1% to poultry. The migratory bird population mainly comprises families belonging to the orders *Anseriformes* and *Charadriformes*. We highlight the importance of epidemiological surveillance systems and the possible role of different migratory birds in the transmission of AIVs within the Americas. Our findings demonstrate the limited information on AIVs in Latin America and highlight the need of more studies on AIVs at the regional level, particularly those focused on identifying the endemic subtypes in regional wild birds.

## Introduction

Avian influenza or bird flu is a highly contagious acute viral disease that can occur in epidemics and cross-border forms in poultry. Influenza A viruses are the aetiological agent of avian influenza and belong to the *Orthomyxoviridae* family. Influenza viruses also includes types B, C [1], and D [2] viruses; however, there is no evidence that type B, C, and D can infect avian species [3, 4]. The natural reservoir of influenza A viruses are avian species within the orders *Anseriformes* and *Charadriformes* [3]. At least 16 of the 18 known haemagglutinin subtypes (H1-H16) and 9 of the known neuraminidase (N1-N9) subtypes have been identified in avian species [1]. Additionally, influenza A viruses can also infect different mammal species including humans, horses, pigs, cats, dogs, and even some marine mammals [5–7]. Furthermore, a new lineage of influenza A viruses have been recently identified in bats in Guatemala and Peru [8, 9], suggesting the existence of other natural reservoirs of the virus. Nevertheless, the mechanisms that allow some influenza A viruses to cross the interspecies barrier are not clearly understood [10].

Influenza A viruses are pleomorphic, enveloped, and contain 8 genomic segments of negative-sense single strand RNAs (-ssRNA) [1, 11]. The high genetic variability of this virus is the result of its mutagenic capacity (antigenic drift) and its potential to exchange genetic segments when two or more viruses infect the same cell (antigenic shift) [1]. These mechanisms of viral diversification have allowed the emergence of new variants, some with zoonotic and pandemic potential, hindering prevention, control, and treatment [7]. Additionally, these genetic changes may be associated with patterns of infection (e.g., epidemic or pandemic) [12] and the course of the disease (morbidity and mortality rates) [13, 14].

From the pathogenic point of view, influenza A viruses in birds are classified as highly pathogenic (HPAI) or low pathogenic (LPAI) avian influenza viruses. To date, only the H5 and H7 subtypes have been proven to be HPAI viruses, although not all H5 and H7 viruses are HPAI viruses [10, 15, 16]. However, LPAI H5 or H7 viruses may become HPAI viruses due to mutations that occur after infection of poultry [10]. The presence of influenza viruses in poultry has serious repercussions on animal health, public health and trade of live poultry or poultry products [13]. Due to some influenza A viruses are zoonotic and zoonotic influenza infections are a pandemic threat, all influenza A viruses found in poultry need to be notified to the World Organisation for Animal Health (OIE). It is estimated that HPAI viruses have greatly affected avian health and poultry production worldwide. More than 500 million poultry deaths have been associated with avian influenza infections [17, 18] producing economic, political and/or sociocultural repercussions [19].

The importance of poultry in the economy of Latin America has increased in recent years [20] and the risk of a possible transcontinental outbreak of avian influenza is a reality. There is a large number of live animals and poultry products moved within and between Latin-American countries [21, 22]. Hence an outbreak of HPAI in Latin America could cost more than USD $1.632 billion, which may represent 0.09% of the gross domestic product in the region [21, 22].

The economic losses from HPAI outbreaks in Asia reached devastating numbers. In 2003 the HPAI outbreak left losses over $55 million USD (45 million dead/slaughtered animals) in Vietnam, and of $387 million USD (16.2 million dead animals) in Indonesia [22]. In the Americas, the economic losses associated with outbreaks of HPAI surpassed $31 million USD in Chile in 2002, with more than 635,000 dead or slaughtered birds [23], and reached approximately $475 million USD in Mexico in 2012, with more than 22.4 million slaughtered birds [24].

Moreover, in the Americas, there is a constant and unavoidable risk of transmission of avian influenza due to the migration of wild birds between the continents [17]. This natural behaviour of some avian species represents one of the most important challenges for programmes to prevent and control of AIVs. Bird migration in Latin America involves 500 to 1000 million birds of more than 42 species. Some avian species are the natural reservoir for AIVs and their natural migration represent a potential route of transmission of AIVs for local wild and domestic species [25, 26]. As a result of avian migration, a wide variety of wild birds carrying avian influenza could arrive from Northern countries, where AIVs are commonly found and where the genetic variability reported for the virus is higher than in the South American continent [12, 27]. This phenomenon of viral spread throughout migration routes has been shown for other viral diseases that affect birds. For example, there is enough epidemiological and phylogenetic evidence that indicates that once the West Nile Virus emerged in North America it gradually moved towards South America through migratory wild birds, spreading from Canada to Argentina in just seven years [28–30].

The circulation and consequences of AIVs in Latin American have been documented before [31, 32]. However, this information is limited if we take into account that some of the countries within the region hold the greatest diversity of avian species in the world [33–35]. Furthermore, to our knowledge there is no published studies collecting updated information on avian influenza viruses in Latin America. Therefore, it is important to identify areas of knowledge that must be strengthened to develop regional programmes for the prevention, control, and eradication of AIVs. Thus, the objective of this study was to systematically review the published literature on molecular or serological diagnostic of AIVs in birds in Latin America. We found that several AIVs subtypes have been identified throughout Latin America and that these viruses are usually related phylogenetically to North American AIVs. In addition, our results support the hypothesis that AIVs could spread across the Americas through avian migration becoming a constant threat for the poultry industry in the region.

## Materials and methods

### Data collection and inclusion criteria

A retrospective study was conducted to systematically review the peer reviewed articles on avian influenza in Latin America published from January 2000 to December 2015. We followed the PRISMA statement (*Preferred Reporting Items for Systematic Reviews and Meta-Analyses—2010*) [36] and the STROME ID guide (*Strengthening the Reporting of Molecular Epidemiology for Infectious Diseases-2014*) [37]. The systematic review included all serological and molecular studies on influenza in birds in Latin America (i.e., all countries in the Americas, except the USA and Canada) as the main inclusion criteria. The articles were obtained from the MedLine/PubMed (http://www.ncbi.nlm.nih.gov/PubMed) and SciELO-Scientific Electronic Library Online (http://www.scielo.org/) databases. For the systematization of information, a database was built that included the references of all selected publications, as well as the title, author, year of publication, country or countries where the study was conducted, collaborating countries, language of publication. From the results, we looked for diagnostic methodology (serological or molecular), percentage of positive samples, viral subtypes diagnosed and study population divided into local birds (both poultry and wild) and migratory birds (aquatic and non-aquatic).

### Exclusion criteria

Notes, letters to the editor, news, editorials, case reports in humans, and experimental inoculation studies were manually excluded. Case reports in humans, or other species different to birds, reports that did not belong to Latin America, or were not serological or molecular studies were also excluded. Phylogenetic and other molecular studies of previously reported AIVs were excluded.

### Search strategy

The processes for searching, selecting, and collecting the articles in the databases were conducted using MeSH keywords and Boolean connectors. The “Limits” function included in the search engines was used to define the specific years and began with a general search at the level of Latin America. Subsequently, a more detailed search was performed for each individual country.

One example search is: (“influenza in birds”[MeSH Terms] OR (“influenza”[All Fields] AND “birds”[All Fields]) OR “influenza in birds”[All Fields] OR (“avian”[All Fields] AND “influenza”[All Fields]) OR “avian influenza”[All Fields]) AND (“Latin America”[MeSH Terms] OR (“Latin”[All Fields] AND “America”[All Fields]) OR “Latin America”[All Fields]).

The articles were selected using a two-stage approach. During the first stage, the publications were selected based on their titles and abstracts, excluding the publications that were not considered relevant for this study. During the second stage, the full text of the articles that were previously selected in the first phase was reviewed. At this point, the articles that did not meet the previously established criteria were excluded. As previously reported [38], two researchers independently selected the publications and filtered the information to enhance the methodological strength, and disagreements were resolved during public discussions with the research team.

### Assessment of the quality and risk of bias of the included studies

Due to the big amount of genomic data of AIVs and the use of the STROME ID guide, the quality of the studies and risk of bias was evaluated according to the criteria of the QUADOMICS—an adaptation of QUADAS, a quality assessment tool used in systematic reviews of diagnostic accuracy studies—was developed to assess quality issues specific to ‘-omics’ research, including the quality assessment of studies included in systematic reviews [39]. The methodologies of studies that achieved 12/16 or more on the QUADOMICS tool could be classified as ‘high quality’, whereas those that scored 11/16 or lower must be classified as ‘low quality’. Two different researchers double-checked the general characteristics and methodological quality assessment independently.

### Statistical analysis

Descriptive statistics were used in all cases to evaluate the results (frequencies and percentages). To examine the frequency of AIVs studies in Latin America over time, the analysed period was divided into quartiles, and presented as absolute percentage over the total studies. Statistical analyses were performed using GraphPad Prism^®^ 7 for Windows^®^ (GraphPad Software, San Diego California USA).

## Results

**Fig 1:**
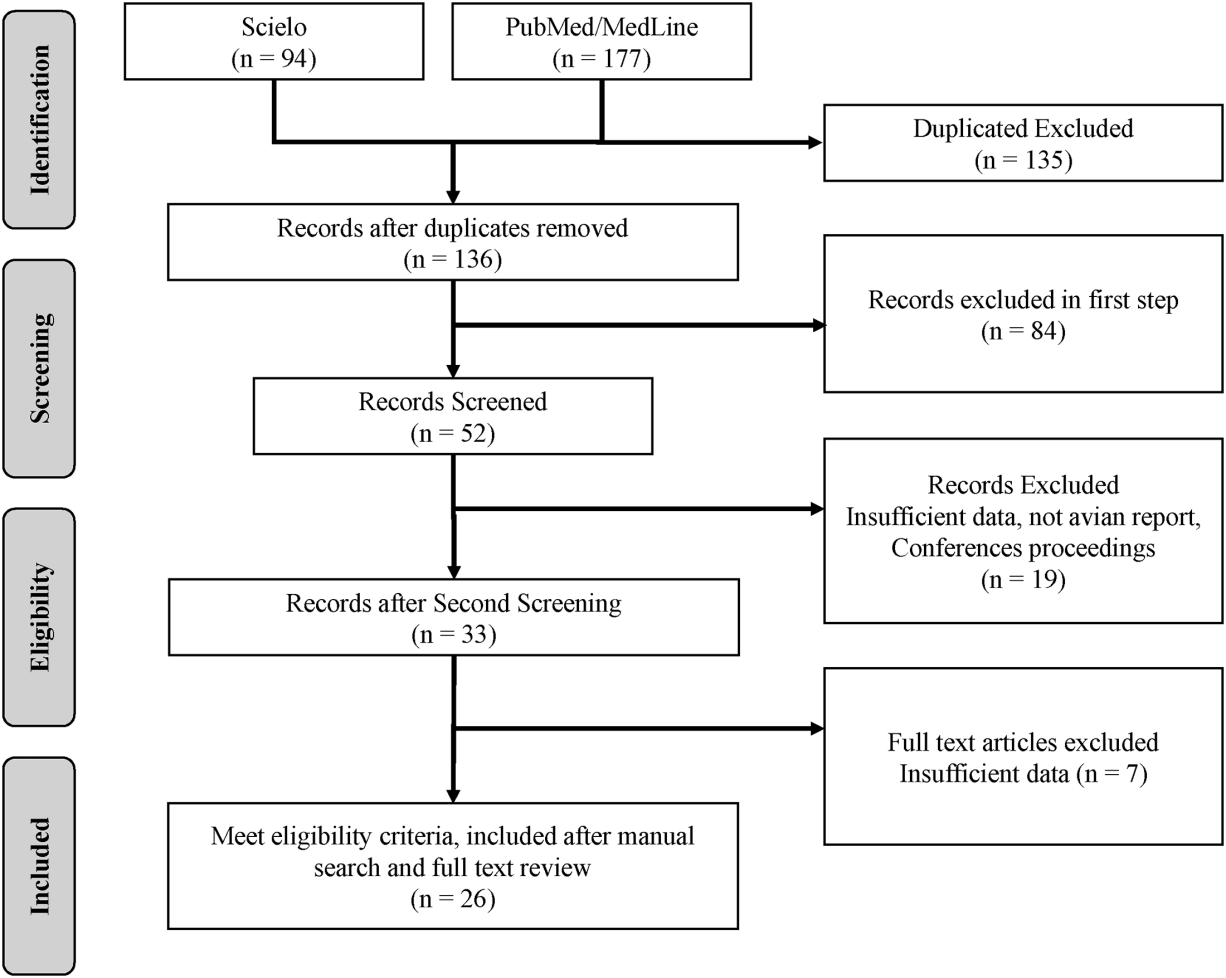
PRISMA diagram. Summary of the literature search.

Two hundred and seventy one peer reviewed articles were initially found from the two selected databases; 49.8% (n = 135) of these were duplicates and were excluded during the first selection phase. Of the remaining 136 references, 61.8% (n = 84) did not meet the inclusion criteria of the study and were also excluded. After reviewing the full text of the remaining articles (n = 52), only 50% (n = 26) met all the inclusion criteria for this study (Fig 1 –S1 Table). Of these 26 studies, 92.3% (n = 24) were published in English, 7.7% (n = 2) were published in Spanish, and none were published in Portuguese.

We conducted quality assessment according to QUADOMICS, The results showed that almost 77% of the studies (n = 20) were classified as “high quality.” The remaining 23% (n = 6) were classified as “low quality.” (S2 Table). None of the studies stated whether the index test results were interpreted without knowledge of the results of the reference standard and the converse, thus failing criteria 12 and 13 of the QUADOMICS tool. That means that most of the studies included in the systematic review has sufficient information for the systematic analysis.

**Fig 2:**
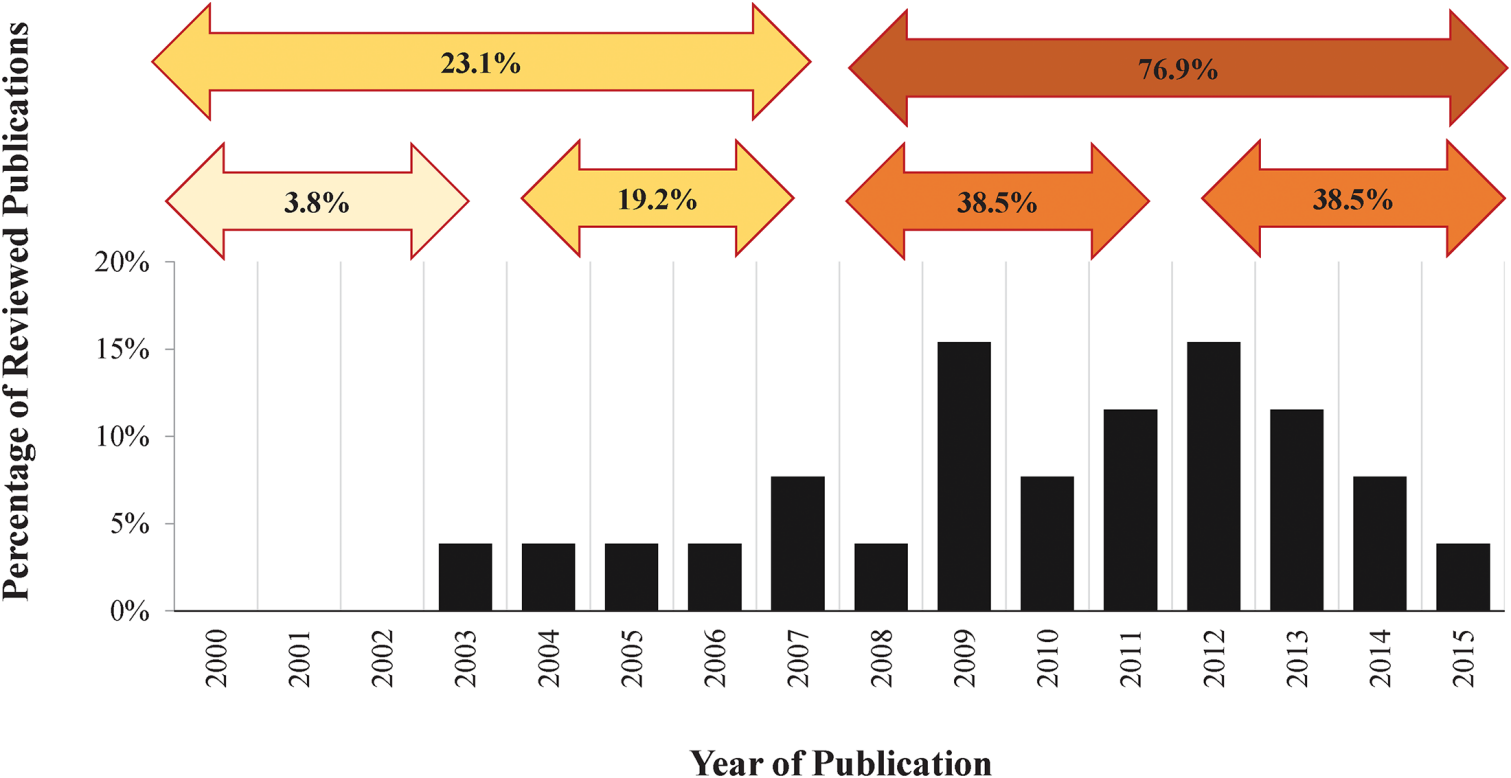
Avian influenza publication trends. Frequency distribution of published serological and molecular studies of avian influenza in Latin America by year. Upper arrows indicates the frequency of AIVs studies in Latin America over time (quartiles).

Of the articles analysed, 23.07% (n = 6) were exclusively published by Latin American authors [40–45], whereas the remaining 76.93% (n = 20) were the result of interinstitutional collaborations. The United States of America was the country that cooperated the most in studies on AIVs in Latin America (88.5%, n = 18), followed by the United Kingdom (3.8%, n = 1) and Spain (3.8%, n = 1). The period analysed was divided into quartiles to examine the frequency of AIVs studies in Latin America over time. This analysis showed that 76.9% (n = 20) of the articles were published during the third and four quartile, from 2008 to 2015, whereas only 23.1% (n = 6) of the articles analysed were published during the first and second quartile (from 2000 to 2007). The largest number of publications appeared in 2009 (n = 4) and 2012 (n = 4) (Fig 2).

**Fig 3:**
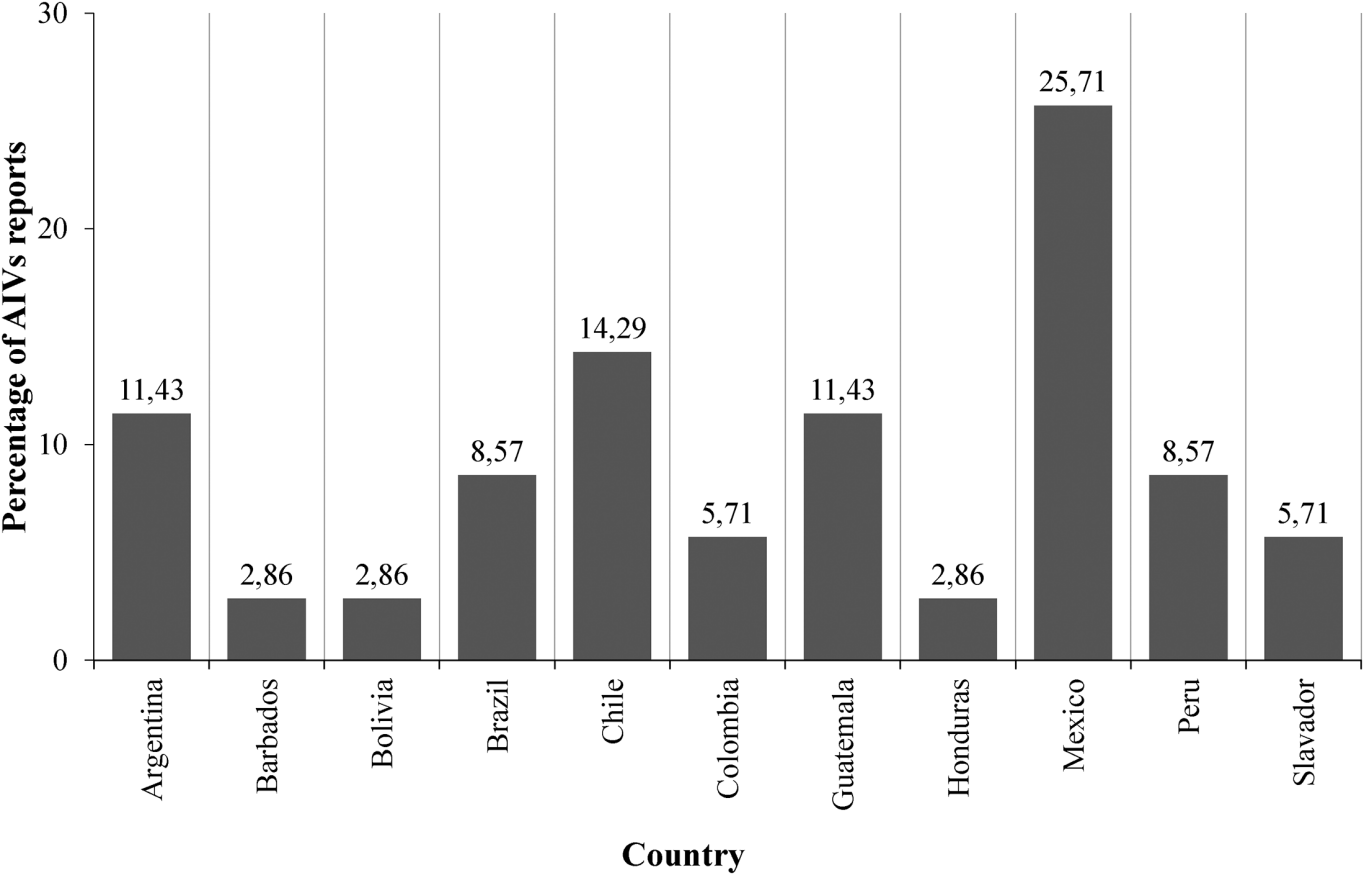
Avian influenza reports by country. Percentage distribution of avian influenza reports by country from 2000–2015.

There were publications on AIVs originating in most Latin American countries. Mexico was the country with the largest number of conducted studies and reported cases during the period analysed, followed by Chile and Argentina (Fig 3). After reviewing the 26 selected publications, a total of 35 published records on the presence of avian influenza in Latin America were identified, which reported the presence of at least 14 different subtypes of influenza in birds, including LPAI (n = 13–92.9%) and HPAI (n = 1–7.1%) subtypes (Table 1). No reported cases of AIVs were found in Venezuela, Paraguay, Uruguay and Ecuador. Also, according to the analysed papers most of the AIVs (n = 17–65,38%) were reported trough surveillance systems in migratory and local birds; case studies and cases reports account only for 26.92% (n = 7) and 7.69% (n = 2) respectively.

**Table 1:** Identification of different subtypes of avian influenza in birds Latin America. The table shows the viral subtype detected, the species, the country where it was identified, the authors, and the year of publication.

AIVs Subtype	Country	Year of report	Avian Population	Reference
			**Poultry birds**	
H5N2	Guatemala	2002–2003	Chickens *(Gallus gallus)*	[47]
H5N2		2000	Chickens *(Gallus gallus)*	[60]
H7N3	Chile	2002	Chickens *(Gallus gallus)*	[47]
H7N3*		2002	Chickens *(Gallus gallus)*	[46]
pH1N1		2009	Turkeys *(Meleagris Sp)*	[45]
H9N2	Colombia	2005	Chickens *(Gallus gallus)*	[47]
H5N2		2011	Chickens *(Gallus gallus)*	[48]
H5N2		2011	Japanese quail (C*oturnix japonica*)	[48]
H5N2		2011	Domestic duck (*Anas platyrhynchos domestica*)	[48]
H7N3*	Mexico	2012	Chickens *(Gallus gallus)*	[62]
H5N2		2001	Chickens *(Gallus gallus)*	[60]
H5N2	Honduras	2001	Chickens *(Gallus gallus)*	[60]
			**Wild Local Birds**	
H7N3	Bolivia	2001	Cinnamon teal (*Anas cyanoptera*)	[65]
H10N9	Peru	2006	Ruddy turnstone *(Arenaria interpres)*	[49]
H3N8		2006	White-cheeked pintail *(Anas bahamensis)*	[49]
H10N9		2006	American oystercatcher (*Haematopus palliates)*	[49]
H4N5		2007	Peruvian pelican (*Pelecanus occidentalis thagus)*	[49]
H3N8		2006	Cinnamon teal (*Anas cyanoptera)*	[49]
H13N2		2007	Whimbrel (*Numenium phaeopus)*	[49]
H13N2		2007	Dominican gulls (*Larus dominicanus*)	[49]
H12N5		2008	Andean coot (*Fulica ardesiaca*)	[44]
H7N3 / H2N9		2008	White-cheeked Pintail (*Anas bahamensis*)	[57]
H7N3 / H2N9		2008	Cinnamon teal (*Anas cyanoptera*)	[57]
H7N3 / H2N9		2008	Andean duck (*Oxyura ferruginea*)	[57]
H13N9	Argentina	2006	Wild kelp gull (*Larus dominicanus*)	[53]
H5N3		2007–2010	Silver teal (*Anas versicolor*)	[54]
H9N2		2007–2010	Rosy-billed pochards (*Netta peposaca*)	[52, 54]
H6N2		2007–2010	Rosy-billed pochards (*Netta peposaca*)	[54]
H6N8		2007–2010	Rosy-billed pochards (*Netta peposaca*)	[54]
H7N9		2007–2010	Cinnamon teal (*Anas cyanoptera*)	[54]
H1N1		2008	Red-winged tinamou (*Rhynchotus rufescens*)	[50]
H5/H7/H9N?	Brazil	2006	Burrowing owl (*Speotyto cunicularia*)	[51]
H5/H7N?		2006	Barn owl (*Tyto alba*)	[51]
H5/H7/H9N?		2006	Rock pigeon (*Columba livia*)	[51]
H7N?		2006	Ruddy ground dove (*Columbina talpacoti*)	[51]
H7N?		2006	Toco toucan (*Ramphastos toco*)	[51]
H9N?		2006	Campo flicker (*Colaptes campestris*)	[51]
H5/H7/H9N?		2006	Red-legged seriema (*Cariama cristata)*	[51]
H5/H7N?		2006	Black-crowned night-heron *(Nycticorax nycticorax*)	[51]
H5/H7/H9N?		2006	Chalk-browed mockingbird (*Mimus saturninus*)	[51]
H5/H7N?		2006	Guira cuckoo (*Guira guira*)	[51]
H5N2	Colombia	2011	Whistling ducks (*Dendrocygna Sp*)	[48]
			**Migratory Birds**	
H12N5	Peru	2008	Ruddy turnstone (*Arenaria interpres*)	[44]
H11N9	Brazil	2008	Ruddy turnstone (*Arenaria interpres*)	[56]
H3N?		1998	Olivaceous elaenia (*Elaenia mesoleuca*)	[43]
H3N?		1998	Red-eyed vireo *(Vireo olivaceus)*	[43]
H6N?	Mexico	2008	Green-winged teal *(Anas crecca)*	[42]
H9N?		2008	American wigeon *(Anas americana)*	[42]
H5N?		2008	Redhead *(Aythya americana)*	[42]
H5N?		2008	Northern shoveler *(Anas clypeata)*	[42]
H7N3		2006	Cinnamon teal (*Anas cyanoptera*)	[40]
H5N?		2007	Redhead *(Aythya americana*)	[41]
H5N?		2008	Shoveler (*Anas clypeata*)	[41]
H13N2	Chile	2007	Franklin’s gull *(Leucophaeus pipixcan)*	[67]
H13N9		2009	Franklin’s gull *(Leucophaeus pipixcan)*	[67]
H5N9		2008	Kelp gull (*Larus dominicanus*)	[67]
H7N9	Guatemala	2007–2008	Blue-winged teal (*Anas discors*)	[58]
H11N2		2009–2010	Blue-winged teal (*Anas discors*)	[58]
H8N4		2009–2010	Blue-winged teal (*Anas discors*)	[58]
H5N3		2009–2010	Blue-winged teal (*Anas discors*)	[58]
H5N4		2009–2010	Blue-winged teal (*Anas discors*)	[58]
H3N8		2009–2010	Blue-winged teal (*Anas discors*)	[58]
H4N3	Barbados	2004	Blue-winged teal (*Anas discors*)	[55]

* Indicates the presence of HPAI.

### Wild birds vs. poultry

**Fig 4:**
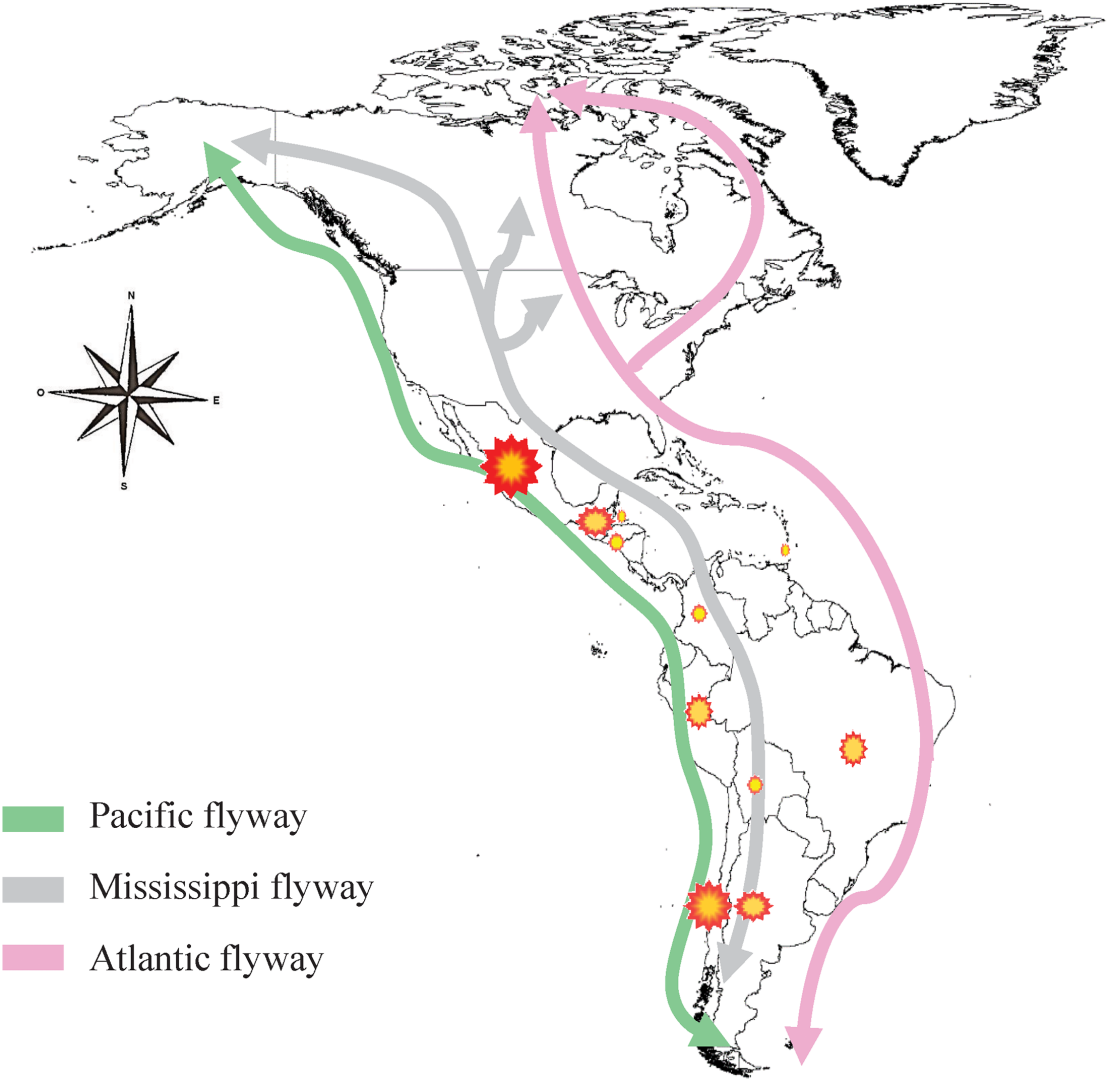
Spatial representation of the reports of avian influenza in Latin America and the migratory routes of birds on the continent. The coloured dots represent the percentage of records of AIVs circulation in different countries.

Of the reported cases of AIVs in Latin America, 43.7% corresponded to migratory birds, 28.1% to local wild birds, and 28.1% to poultry. The migratory bird population mainly comprises families belonging to the orders *Anseriformes* (e.g., ducks, geese, and swans) and *Charadriformes* (e.g., gulls, terns, oystercatchers) (Table 1). Birds of these families migrate from the northern part of the continent to the south using the three main known migratory routes: Pacific, Mississippi, and Atlantic. As shown in Fig 4, the reports on AIVs overlap with the different migration routes. Moreover, broilers [46], laying hens [47], and, to a lesser extent, domestic ducks [48], quail [48], and commercial turkeys [45] were the poultry reported to be affected by avian influenza. In terms of local wild birds, studies mainly include families belonging to the same orders *Anseriformes* (mainly ducks) and *Charadriformes* (mainly represented by seagulls), but there are also reports of avian influenza in pelicans, toucans, owls, and doves, among others [43, 44, 49–51].

### Percentage of positive samples

Studies analysing samples from clinically ill animals that participated in diagnostic programmes in Brazil (27 to 87%) [43, 51], Chile (62%) [45], and Argentina (34.5%) [50] showed the highest percentage of positive samples. The percentage of positive samples recovered during routine surveillance programmes suggested low infection rates, ranging from 0.07% to 0.62% in Argentina [52–54]; 1.19% in Barbados [55]; 0.71% in Brazil [56]; 3.6% in Mexico [41]; and between 0.22% and 0.88% in Peru [44, 57]. However, these results may not represent the actual prevalence rates of avian influenza in the countries analysed.

In some specific cases, such as in Colombia [48] and Guatemala [58], authors have found a 3.6% of positive samples in wild birds and 2.6% in poultry [48]. In general, other studies have found 1.7% of positive samples in wild birds, 11.2% in aquatic birds, and 0.3% in land birds, highlighting the importance of aquatic wild birds in the ecology of AIVs [58].

### Countries with AIVs of high (HPAI) and low (LPAI) pathogenicity

Each country reported showed a different pattern of circulating subtypes over time. For example in Mexico, the LPAI H5N2 virus circulated during the first quartile of the study (2002 to 2004). Then, no reports were published for 4 years. However, between 2009 and 2012, a LPAI H7N3 viruses were initially isolated and then became a HPAI viruses over time as a result of mutations in its genome [59]. Furthermore, in Chile, the first case of AIVs was reported in 2004, corresponding to an outbreak of the LPAI H7N3 subtype that has affected poultry since 2002 and accumulated mutations to become a HPAI virus, one month later in the same year [46].

### Central America and the Caribbean case studies

Most published research studies from Latin American countries located in Central America and the Caribbean were conducted in Mexico, Guatemala, El Salvador, and Honduras. The LPAI H5N2 viruses were identified on multiple occasions from 2000 to 2005 [47, 60] and were phylogenetically linked to similar viruses that circulated in Mexico in the 90s [60]. Moreover, LPAI H7N3 viruses were isolated in Mexico in 2009 from wild duck samples (*Anas cyanoptera*) and were phylogenetically linked to North American viruses [40]. Simultaneously, another study reported the circulation of AIVs of the H5, H6, and H9 subtypes, which belong to North American lineages that are very closely related to virus isolates from California in previous years [42], demonstrating the continuous migration of AIVs throughout the avian migration seasons and suggesting the occurrence of AIVs transmission from resident waterfowl to poultry or other domestic animals [41].

The genomic characterization of a HPAI H7N3 viruses isolated from laying hens in 2012 in Jalisco, Mexico determined that these viruses had high percentage of sequence identity (> 97%) in its eight genomic segments with AIVs from North America specially those isolated from ducks in Mississippi, Missouri, Arkansas, and Illinois [59]. Furthermore, the Food and Agriculture Organization of the United Nations (FAO [61]) estimated the consequences that this outbreak could bring to neighbouring countries if strict border controls are not enforced and biosafety procedures are not guaranteed in the poultry industry. In 2014, Lu *et al* were seeking to understand the phylogenetic and geographic origins of these HPAI H7N3 viruses. Authors found that the virus originated in the North American lineage through genetic reassortment events, whereby five of the eight segments (HA, NA, NP, M, and NS) were introduced by wild birds migrating along the central North American migratory flyway and the PB2, PB1, and PA gene segments were introduced through the Western North American flyway [62].

In El Salvador, Guatemala and Honduras [47, 55, 58, 60, 63], LPAI H5N2 has been repeatedly isolated, with the first reports appearing in Guatemala and El Salvador from 2000 to 2001. Phylogenetic analyses demonstrated that the haemagglutinin sequences of these viruses were related to H5N2 viruses that have been circulating in Mexico since 1994 [47]. However, the genomic diversity of AIVs in the region might be higher (Table 1). In 2004, the H4N3 subtype LPAI virus was reported in Barbados [55], and LPAI H7N9, H11N2, H3N8, H5N3, H8N4, and H5N4 viruses were identified in Guatemala between 2007 and 2010 [58]. In all cases, wild migratory ducks were involved (*Anas discors*—Blue-winged teal), indicating the importance of this avian species in the maintenance and transmission of AIVs in the continent [58]. In addition, phylogenetic analyses revealed that these viruses reported in Central America and the Caribbean were highly similar to the North American lineage of viruses, suggesting that bird migration is strongly correlated to the ecology of these viruses [55, 58, 64].

### Case studies of AIVs in South America

Although two serological studies reported the presence of only H3 AIVs since the late 90s in Brazil,[43] a diversification of anti-AIV antibodies was reported by 2006 and exposure to H5, H7, and H9 subtypes of AIVs in multiple wild bird species was demonstrated (Table 1) [51]. Additionally, a molecular characterization study of AIVs in Brazil reported the presence of the LPAI virus subtype H11N9 in Ruddy turnstone (*Arenaria interpres*). Through phylogenetic analysis, de Araujo *et al*, 2014, concluded that these H11N9 viruses were genetically related to AIVs isolated in the same year from the same species in New Jersey, USA [56].

One of the first isolations of AIVs in South America were LPAI H7N3 viruses recovered from wild aquatic birds, Cinnamon teal (*Anas cyanoptera*), in Bolivia in 2001 [65]. According to the phylogenetic analysis, these viruses were related to viruses subsequently identified in Chile in 2002 [46] and AIVs from the North American lineage of viruses found in wild aquatic birds [65], indicating that there is an important exchange of viral genes between North and South America AIVs.

In Colombia, AIVs were first detected in 2005 through the serological detection of antibodies against H9N2 in broilers [47]; however, no H9N2 has been isolated in the country. Therefore, no additional information is available about the circulating virus reported in that study [47, 66]. Subsequently, serological and molecular evidence demonstrated the circulation of LPAI H5N2 viruses among Colombian wild birds from 2010 to 2012 [48]. These H5N2 viruses were identified in samples taken from different species of wild birds from the *Llanos Orientales* region (Table 1). Furthermore, these Colombian viruses were at least 96% identical, at the HA and NA genes, to the North American AIVs identified in wild migratory ducks [48].

In Peru, seven LPAI subtype were reported from 2009 to 2012, five of which (H2N9, H4N5, H10N9, H12N5, and H13N2) have not been reported in other Latin American countries [44, 49, 57]. As in other studies, it was concluded that the presence of these AIVs in local wild birds constitutes a risk for economically important species in the region and that circulating viruses are closely related to North American lineage viruses [44].

Chile is one of the countries with the greatest diversity of AIVs reported. AIVs were first reported in Chile in 2002 [46, 47] and included LPAI and HPAI H7N3 viruses, reporting for the first time, the emergence of HPAI in South American. LPAI H7N3 viruses were the first to appear, affecting a batch of broiler breeders; however, two months later, an increased rate of mortality was observed in these birds due to genetic changes that resulted in a sharp variation in both the virulence and pathogenicity of the outbreak [46]. In this case, a haemagglutinin insertion of 10 amino acids at the cleavage site allowed the H7N3 LPAI to become HPAI. Although this Chilean viruses were related to North American strains, phylogenetic analyses demonstrated that these Chilean H7N3 viruses formed a monophyletic clade diverging from all the H7N3 viruses reported in GenBank, [46]. Due to the emergence of AIVs in Chile, the Servicio Agrícola y Ganadero de Chile (Agricultural and Livestock Service of Chile) established a sentinel surveillance program and found LPAI H13N2, H5N9, and H13N9 AIVs in seagulls (Table 1) between 2007 and 2009. All LPAI found in Chile have been phylogenetically associated to North American strains recovered from wild birds [67].

In Argentina, all AIVs publications include low pathogenic subtypes (n = 4) and none HPAI. Between 2006 and 2007, a sentinel surveillance program identified AIVs in wild seagulls (*Larus dominicanus*). In this study, samples were collected on the Atlantic coast of southern Argentina, and resulted in the molecular characterization of an H13N9 AIV. Phylogenetic analysis of the 8 gene segments of these viruses showed that the 6 internal gene segments were phylogenetically related to viruses isolated in Chile and Bolivia in previous years, indicating a pattern of regional genetic evolution with limited reassortment events [53]. Nevertheless a year later (2008), LPAI H1N1 viruses were diagnosed in local birds (Red-winged tinamou—*Rhynchotus rufescens*) at the coastal region of the Rio de la Plata, which were found dead [50].

In 2007 in Argentina, an H9N2 AIVs was first isolated from wild aquatic birds (*Netta peposaca—*Table 1) [52] and another study confirmed the presence of the H5N3, H9N2, H6N2, H6N8, and H7N9 subtypes between 2007 and 2010 (Table 1) [54]. Phylogenetic analyses of the different viral segments showed that those South American lineage of AIVs, conserve an independent evolutionary pathway, but also have an evolutionary genetic relationship with the North American AIVs lineage, suggesting the presence of a common ancestor [52, 54].

### Pandemic influenza virus in Latin American birds

The H1N1 pandemic strain (pH1N1) has only been reported in breeding turkeys from two farms in Valparaiso (Chile) in July 2009, where it caused mild infections, reproductive symptoms and a gradual process of natural recovery [45, 68]. Although this virus is not considered an AIVs, its presence in poultry increases the risks and potential losses for an industry of great importance in the region and demonstrates the inter species transmission of some type A influenza viruses.

## Discussion

Studies on AIVs in Latin America are limited, although several AIVs subtypes have been identified in Latin American countries since the 90s [69–71], however, the diversity, distribution, and potential impact on the poultry industry is poorly understood. In this systematic review, we present the scientific evidence on AIVs in Latin America published in the first 15 years of the 21^st^ century. We found an increased number of studies on AIVs over time due to better regional surveillance systems that aimed for early diagnosis to prevent the spread of HPAI strains [46, 68]. However, in the study period, only 26 published articles met the inclusion criteria of our review, and illustrated the paucity of AIVs research in Latin America compared to other areas of the world [72]. Therefore, in the near future it is imperative to characterize the molecular diversity and epidemiology of AIVs at the regional level to develop health interventions that minimize the risk of transmission within birds and from birds to other animal species including humans.

One of the most important results of this systematic review is the consolidation of information on AIVs subtypes that have circulated in Latin America. Furthermore, the scientific studies on these viruses in Latin America and their phylogenetic relationships with North American viruses, demonstrate the transboundary movement of AIVs within the Americas possibly associated to natural migration of avian species [57, 58]. However, other major routes of viral circulation through the (legal or illegal) trade of live birds or poultry products cannot be ruled out because these routes have also been shown to be important channels for the transcontinental spread of AIVs [27, 43]. Therefore, further research on the molecular epidemiology of AIVs in the Americas is needed to identify the most likely routes of transmission and possible risk of virus movements within and between countries.

Worldwide, wild aquatic birds are the natural reservoirs of all AIVs [47, 60, 64]. Hence, it was expected that wild aquatic birds in Latin America and migratory aquatic birds from the United States and Canada harboured AIVs [40] as we found. During their migration, most of these birds inhabit wetlands in Colombia, Venezuela, Suriname, Brazil, and Mexico [25, 26]. These wetlands, which are represented by swamps, lagoons, and coasts, are congregation sites for resident and migratory populations [27], which could potentially allow AIVs transmission between migratory and resident birds. Therefore, an expected finding of this systematic review was evidence of an overlap between reports on AIVs in Latin America and the different migratory routes used by birds over North and South America, highlighting the role of migratory species in the epidemiology and diversity of AIVs. However, there is no epidemiological surveillance systems for all Latin America that allows the integration of all collected information in different countries, resulting in a limited interpretation of results at the regional level.

It is possible to assume that the wide genetic variety described in countries like Guatemala is linked to the spatial location of this country, as it is situated in a “geographical bottleneck” (Central American isthmus) that funnels millions of birds migrating along the North American Mississippi, Pacific, and Atlantic flyways [58]. Nevertheless, the genetic diversity of AIVs in this region may be greatly underestimated and should be further investigated.

In addition, analyses of AIVs isolated from *Anas cyanoptera* in Bolivia [65] and from *Larus dominicanus* in Argentina [53, 54] suggest a regional lineage that has evolved locally and had diverged from North American strains. The local evolution of AIVs should be further investigated because it could be explained by an ecological interconnection between different countries and wetlands in Latin America which might be associated with bird migration [53].

It is important to highlight that phylogenetic analyses of AIVs in Latin America has demonstrated their relationships with viruses previously isolated in North America. These findings suggest that there is viral transmission between the Americas [60, 64, 65] and that this transmission could be related to specific migratory flyways. This phenomenon has also been described for the North American and Eurasian lineages [73, 74], strengthening the evidence showing that migratory birds play a key role in the ecology of AIVs. However, additional studies are needed to integrate knowledge about avian migrations with current and future molecular findings on AIVs in the region. This information is crucial to a better understanding the dynamics of AIVs transmission among susceptible populations, both migratory and local, as wild or domestic species.

Although none of the studies reported the presence of avian influenza viruses or genes from the Eurasian lineage during the period evaluated (2000–2015), the recent incursion of an Eurasian H5 subtype HPAI virus in North America [75] highlights the importance of better epidemiological monitoring system to allow their timely detection in Latin America. A paper published after our study period [76] demonstrated for the first time the presence of AIVs genes that are closely related to the Eurasian lineage in Latin America. This recent publication demonstrates the importance of studying AIVs evolution in the region, because the risk of new AIVs emergence in Latin America [76].

Timely identification of disease and mortality events in wild birds is extremely important for early detection of AIVs [1, 61]. Hence it is critical to strengthen AIVs surveillance systems in the region that are supported by multiple non-governmental organizations and the regional governments themselves [77]. This review found that most studies that found higher diversity among AIVs were the result of extensive and constant epidemiological surveillance efforts focused on wild local and migratory birds [43, 49, 53, 56]. However, epidemiological surveillance systems for AIVs in Latin America are weak although they are improving recently in some countries. Additionally, it is important to develop mechanism to prevent and respond to possible AIVs outbreaks. Furthermore, it is crucial to understand and educate on the importance of biosecurity for the poultry industry and backyard poultry production to minimize the risk of transmission of AIVs between wildlife (natural reservoirs) and poultry (susceptible hosts).

Biosecurity measures to confront outbreaks of HPAI viruses have proven to be the most effective strategy to reduce the spread of the virus and the disease in countries in Europe and Asia [78, 79], that’s why the establishment of effective surveillance systems could allow the early identification of emergent AIVs, reducing the impact of a possible AIVs outbreaks in poultry. It is also well know that strong preparedness plans against AIVs outbreaks can minimize its economic and public health impact [80], also those preparedness help to identify gaps and to expand resources where necessary, in order to be better prepared to reduce impact of outbreaks [81].

It has been established that HPAI viruses have originated from LPAI genetic precursor viruses of wild origin that have managed to infect poultry and mutate [74, 82]. In Latin America this events (from LPAI to HPAI) were observed in Chile and Mexico [46, 62]. Although there is no scientific reports on the persistence of these HPAI viruses at the population level, it is necessary to evaluate what happened to these viruses from a biological point of view, whether they were extinguished or could be circulating without being detected. By now, we already know that HPAI H7N3 has been isolated recently in Mexico in 2016 and 2017 as in wild birds as in commercial birds, however the biologic and/or phylogenetic origin of this virus has not been studied yet [83, 84]. This information is crucial because HPAI viruses pose a risk not only for avian farms, but also for public health [85, 86].

Although virulence is a polygenic trait in AIVs, one direct virulence factor is linked to the haemagglutinin cleavage site [1]. In all AIVs, the haemagglutinin is synthesized as a precursor that requires posttranslational cleavage by host proteases before it is functional and the viral particles become infective [11]. All HPAI viruses examined to date have multiple basic amino acid motifs (arginine and lysine) at the haemagglutinin cleavage site; in contrast, the cleavage motifs in LPAI viruses have only two basic amino acids [87]. This difference causes the replication of LPAI viruses to be restricted to sites where trypsin-type enzymes are found (intestinal and respiratory tracts), whereas HPAI viruses are able to replicate systemically, damaging vital organs and tissues and resulting in serious illness that can lead to death [88]. Although it is unclear which biological mechanism explains the acquisition of the multiple basic amino acid motifs by the two strains of HPAI virus reported in Latin America, it is clear that they comply fully with the molecular and biological requirements to be classified as HPAI viruses [46, 62].

### Strengths and weaknesses of the systematic review

Systematic reviews allow the revision of all the scientific evidence on a given topic avoiding selection bias. Using this process, the summarized information can be presented to propose hypotheses that explain the behaviour of the data and to identify areas of knowledge where further research is needed [36]. In this study, the current knowledge on AIVs in Latin America was reviewed and several gaps of knowledge were identified. In addition, our review illustrates the strengthening of links between Latin American and non-Latin American research groups over time, which in a globalized world is required to strength science and is consistent with another bibliometric analysis published recently [38]. In our study, inter-institutional cooperation was observed in more than 80% of the publications analysed, a fact that is of great importance because multinational efforts are needed to understand the complexity of the ecology and epidemiology of AIVs [89]. Moreover, the fact that some countries have established AIVs epidemiological surveillance systems for both migratory wild birds and poultry was also seen as a strength [43, 49, 53, 56]. These systems have broadened the knowledge base about these viruses in the region and allowed the establishment of early prevention and possible control steps to prevent the possible spread of AIVs to local birds and poultry species, thus avoiding direct and indirect potential economic losses.

As required by PRISMA and the STROME-ID statement, we also recognize the limitations of this study (S3 Table). Our methods did not allow us to identify significant differences in the identification of AIVs among Latin American countries. However, this is associated to the limited number studies found on the topic reviewed and reflects the need for additional AIVs research in Latin America, not a methodological bias associated with the databases used, due to particularly, those databases are considered the most important locally (SciELO) and globally (PubMed/Medline) and they have thematic relevance [90, 91].

It is a fact that the number of peer-reviewed published studies of AIVs in birds in Latin America represents only a small part of the true panorama of the infection (publication bias). The economic resources available for surveillance and research, geopolitical situation and other factors of each country could add different bias that authors could not control. This bias has been critically analysed and conclusions presented has been showed in the light of this important topic as has been determined by others [92].

## Conclusions

The identification of multiple AIVs subtypes in Latin America is a reality that includes both HPAI and LPAI viruses. However, information about these viruses and their associated epidemiology in the region is scarce. It is undeniable that more AIVs studies are needed at the regional level to identify and differentiate endemic subtypes in regional wild birds. Efficient epidemiological surveillance systems are needed also to allow the timely identification of new AIVs introduced in the region. It is necessary to understand the roles of different migratory birds in the spread of AIVs between North and South America. Additionally, it would be important to identify the main risk factors involved in the transmission of AIVs between wild birds and poultry. Furthermore, identify and implement better biosecurity practices to minimize such risk. It is also important to strengthen inter-institutional collaboration at the regional and transnational level to better understand the dynamics of transmission and distribution of the virus, as well as to optimize the use of resources in these countries, where research funds are very limited compared to the funding of developed countries. It is necessary to maximize regional efforts for the early detection of AIVs in both local and migratory wildlife and to maintain biosafety and biocontainment barriers to prevent infection in poultry.

Due to the emergence of new strains and multiple viral subtypes, it is necessary to make adjustments to the circulation analysis and prediction models that have been previously used elsewhere, focusing on the specific conditions of Latin America, both from the point of view of local and migratory wildlife and from that of poultry systems [93]. Finally, it is suggested that basic viral biological and eco-epidemiological studies are needed to understand the natural patterns of circulation of LPAI viruses in Latin America to understand the risks of viral spread and the likelihood that HPAI strains will emerge in the region.

## Supporting information

S1 Table Full database of AIVs selected papers. (XLSX)

S2 Table QUADOMICS assessment. Methodological quality assessment using the QUADOMICS Tool.
(XLSX)

S3 Table PRISMA checklist. (DOC)

## Abbreviations

AIVs: Avian Influenza Viruses
HPAI: highly pathogenic avian influenza viruses
LPAI: low pathogenic avian influenza viruses
OIE: World Organisation for Animal Health
PRISMA: Preferred Reporting Items for Systematic Reviews and Meta-Analyses
QUADAS: Quality Assessment of Diagnostic Accuracy Assessment
QUADOMICS: an adaptation of QUADAS to assess quality issues specific to ‘-omics’ research
STROME: Strengthening the Reporting of Molecular Epidemiology for Infectious Diseases
